# Impact of histologic hepatic steatosis on virological response to nucleos(t)ide analogue therapy in patients with chronic hepatitis B

**DOI:** 10.1097/MD.0000000000051074

**Published:** 2026-10-09

**Authors:** Sedat Çiçek, Jehat Kiliç, Abdullah Mübin Özercan, Abdulvahap Hohluoğlu, Furkan Kirsoy, Selman Çetin, Ali Çağri Oral, İbrahim Hanifi Özercan, Mehmet Yalniz, İbrahim Halil Bahçecioğlu

**Affiliations:** a Department of Gastroenterology, Firat University Faculty of Medicine, Elaziğ, Türkiye; b Department of Rheumatology, Firat University Faculty of Medicine, Elaziğ, Türkiye; c Department of Pathology, Firat University Faculty of Medicine, Elaziğ, Türkiye.

**Keywords:** antiviral therapy, chronic hepatitis B, HBV DNA suppression, hepatic steatosis

## Abstract

Chronic hepatitis B remains a major cause of cirrhosis and hepatocellular carcinoma worldwide. We investigated whether histologic hepatic steatosis affects hepatitis B virus deoxyribonucleic acid (HBV DNA) suppression in patients receiving antiviral therapy. This retrospective observational study included 307 adult patients with chronic hepatitis B who received tenofovir or entecavir therapy and underwent pretreatment liver biopsy. Demographic, laboratory, virological, radiological, and histopathological data were retrieved from the electronic medical record system. Laboratory parameters included complete blood count, liver function tests, alpha-fetoprotein, renal function tests, and lipid profile. Histopathological evaluation included fibrosis stage, histologic activity index, and hepatic steatosis percentage. Ultrasonographic findings included hepatic steatosis, liver echotexture, and contour. The primary outcome was HBV DNA suppression at 12 months. Long-term virological response was evaluated as time to first HBV DNA suppression during follow-up (maximum 60 months). A total of 307 patients receiving entecavir or tenofovir were included, of whom 258 (84.0%) achieved HBV DNA suppression at 12 months. Patients without suppression had higher baseline aspartate aminotransferase, histologic activity index, histologic steatosis, and baseline log_10_ HBV DNA levels (all *P* < .05). Histologic steatosis ≥5% was more frequent in patients without suppression (42.9% vs 23.6%, *P* = .005). In multivariable analysis, steatosis ≥5% (odds ratio 0.365, 95% confidence interval [CI] 0.179–0.746; *P* = .006) and higher baseline log_10_ HBV DNA (odds ratio 0.842, 95% CI 0.751–0.944; *P* = .003) were associated with a lower likelihood of 12-month suppression, with similar findings in the parsimonious sensitivity model. Kaplan–Meier analysis showed delayed suppression with steatosis (log-rank *P* = .016); however, this association was not significant in the conventional adjusted Cox model (hazard ratio 0.900, 95% CI 0.689–1.175; *P* = .438), and a significant time-dependent interaction indicated non-proportionality (*P* < .001). Histologic steatosis and higher baseline log_10_ HBV DNA were associated with a lower likelihood of 12-month HBV DNA suppression. However, an independent longitudinal association with time to suppression was not demonstrated. Residual confounding by unmeasured metabolic factors cannot be excluded.

## 1. Introduction

Chronic hepatitis B (CHB) remains a major global public health problem, affecting approximately 254 million people worldwide and accounting for nearly 1.1 million deaths annually due to cirrhosis, liver failure, and hepatocellular carcinoma.^[1]^ Long-term suppression of hepatitis B virus (HBV) replication is the primary goal of antiviral treatment because sustained virological control reduces hepatic inflammation, delays fibrosis progression, and lowers the risk of liver-related complications.^[2]^

Potent nucleos(t)ide analogues, particularly tenofovir and entecavir, have become the standard first-line therapies for CHB because of their high antiviral efficacy and favorable resistance profiles. Despite excellent overall treatment outcomes, considerable variability exists in the timing and magnitude of virological response among patients receiving antiviral therapy.^[3]^ Identification of clinical, laboratory, and histopathological factors associated with treatment response may improve risk stratification and optimize patient management.

Hepatic steatosis is increasingly encountered in patients with CHB due to the rising prevalence of obesity, metabolic syndrome, and type 2 diabetes mellitus worldwide. Previous studies have reported hepatic steatosis in approximately 20% to 35% of patients with CHB, although prevalence varies according to population characteristics and diagnostic methods.^[4]^ The interaction between HBV infection and hepatic steatosis remains incompletely understood. While some studies have suggested that steatosis may inhibit HBV replication and promote hepatitis B surface antigen (HBsAg) clearance, others have reported adverse effects on liver disease progression and treatment outcomes.^[5,6]^

The impact of hepatic steatosis on antiviral treatment response is particularly controversial. Several investigations have demonstrated lower rates of virological suppression and delayed treatment response among patients with steatosis, whereas other studies failed to identify an independent association after adjustment for demographic, virological, and metabolic factors.^[7,8]^ Furthermore, the influence of histologically confirmed steatosis on long-term virological outcomes remains insufficiently characterized.

Therefore, the present study aimed to evaluate the association between hepatic steatosis and hepatitis B virus deoxyribonucleic acid (HBV DNA) suppression in a cohort of patients with CHB treated with tenofovir or entecavir. In addition to assessing virological suppression at 12 months, we investigated long-term time-to-suppression outcomes using survival analyses and examined demographic, laboratory, ultrasonographic, and histopathological predictors of treatment response.

## 2. Materials methods

### 2.1. Study design and patient population

This retrospective observational study included 307 consecutive patients with CHB who were followed at our institution and received antiviral treatment with either tenofovir or entecavir between January 2010 and December 2016. Medical records, laboratory findings, virological data, radiological examinations, and liver biopsy results were reviewed retrospectively. Ethical approval for this study was obtained from the Firat University Non-Interventional Research Ethics Committee (approval no. 2025/08-18, 22 May, 2025). The study was conducted in accordance with the Declaration of Helsinki.

**Inclusion Criteria: Patients were** included if they were aged ≥18 years, had chronic hepatitis B (HBsAg positivity for >6 months), underwent pretreatment liver biopsy, received tenofovir or entecavir therapy, and had available baseline and follow-up HBV DNA measurements with at least 6 months of follow-up.

**Exclusion Criteria:** Patients were excluded if they had incomplete clinical, laboratory, virological, or histopathological data; follow-up shorter than 6 months; coinfection with hepatitis C virus, hepatitis D virus, or human immunodeficiency virus; other chronic liver diseases; previous liver transplantation; malignancy; immunosuppressive therapy; or inadequate liver biopsy specimens.

### 2.2. Clinical, laboratory, and histopathological assessment

Demographic characteristics, antiviral treatment type, laboratory parameters, virological markers, imaging findings, and histopathological data were retrospectively obtained from the hospital electronic medical record system (ENLIL, Ministry of Health Information Management System, Türkiye). Laboratory parameters included hemoglobin, hematocrit, neutrophil count, lymphocyte count, platelet count, glucose, albumin, total bilirubin, alanine aminotransferase (ALT), aspartate aminotransferase (AST), alkaline phosphatase (ALP), gamma-glutamyl transferase (GGT), alpha-fetoprotein (AFP), urea, creatinine, total cholesterol, low-density lipoprotein (LDL) cholesterol, high-density lipoprotein cholesterol, and triglyceride levels. Virological assessments included hepatitis B e antigen (HBeAg), anti-HBe, and HBV DNA levels at baseline and during follow-up.

Histopathological evaluation included fibrosis stage, histologic activity index (HAI), and hepatic steatosis percentage obtained from pretreatment liver biopsy specimens. All liver biopsy specimens were independently evaluated by 2 experienced liver pathologists who were blinded to the patients’ clinical and virological data. Histological activity was assessed using the HAI, and fibrosis was graded according to established staging systems. Hepatic steatosis was recorded as the percentage of hepatocytes containing fat droplets. No statistically significant differences were observed between the evaluations of the 2 pathologists, and the consensus histopathological findings were used for the final analyses. For subgroup analyses, histologic steatosis was categorized as <5% and ≥5%, consistent with current international histopathological criteria for hepatic steatosis.^[9]^

Ultrasonographic findings obtained at baseline were reviewed from radiology reports. Hepatic steatosis was classified as absent or present according to routine ultrasonographic evaluation. Liver parenchymal echotexture was categorized as normal, fine granular, or coarse granular. Liver contour was classified as normal, irregular, or nodular. These ultrasonographic parameters were recorded and analyzed for their potential association with virological response and HBV DNA suppression during follow-up.

### 2.3. Outcome definition

The primary outcome was virological suppression at 12 months after antiviral treatment initiation. Serum HBV DNA levels were measured using the cobas HBV assay on the cobas 6800/8800 System (Roche Molecular Systems, Branchburg), with a lower limit of quantification of 10 international unit (IU)/mL. Virological suppression was defined as an HBV DNA level below 10 IU/mL, including both undetectable and detectable but below the lower limit of quantification results. Undetectable HBV DNA results were recorded as such and were not assigned a numerical value of 0 IU/mL. HBV DNA measurements were routinely performed at baseline and at approximately 6-month intervals during antiviral treatment. For the 12-month analysis, patients were classified according to the presence or absence of virological suppression based on the HBV DNA measurement obtained closest to 12 months within a predefined window of 10 to 14 months after treatment initiation.

In addition, long-term virological response was evaluated using time-to-event analyses. For these analyses, the event was defined as the first documented HBV DNA level <10 IU/mL during follow-up. Time to virological suppression was calculated from the initiation of antiviral treatment to the first visit at which HBV DNA suppression (<10 IU/mL) was documented. Because baseline HBV DNA levels were positively skewed, HBV DNA values were log_10_-transformed (log_10_ IU/mL) before statistical analyses. The transformed values were used in the multivariable logistic regression and Cox proportional hazards regression models. Follow-up data were evaluated for up to 60 months. Kaplan–Meier survival analysis and Cox proportional hazards regression were performed to assess factors associated with the timing of HBV DNA suppression. Patients who did not achieve virological suppression during follow-up were censored at their last available visit.

### 2.4. Statistical analysis

Statistical analyses were performed using IBM SPSS Statistics version 26.0 (IBM Corp., Armonk). The distribution of continuous variables was assessed using visual methods (histograms and probability plots) and normality tests. Normally distributed variables were presented as mean ± standard deviation, whereas non-normally distributed variables were expressed as median (minimum–maximum). Comparisons between 2 independent groups were performed using the independent samples *t* test for normally distributed variables and the Mann–Whitney *U* test for non-normally distributed variables. Categorical variables were compared using the Pearson chi-square test or Fisher’s exact test, as appropriate. Results were presented as frequencies and percentages. Variables entered into the multivariable models were selected based on clinical relevance and univariable associations to avoid model overfitting. To identify factors associated with HBV DNA suppression at 12 months, variables with clinical relevance and/or significant associations in univariable analyses were entered into a multivariable binary logistic regression model. Odds ratios (ORs) and 95% confidence intervals (CIs) were calculated. Given the limited number of patients without HBV DNA suppression at 12 months, an additional parsimonious multivariable logistic regression model was performed as a sensitivity analysis to reduce the potential for overfitting. This model included histologic steatosis, baseline log_10_ HBV DNA, HBeAg status, and antiviral treatment. Model calibration was assessed using the Hosmer–Lemeshow goodness-of-fit test, and model discrimination was evaluated by receiver operating characteristic curve analysis with calculation of the area under the curve. Time to HBV DNA suppression was analyzed using Kaplan–Meier survival analysis. Differences between groups were evaluated using the log-rank (Mantel–Cox) test. Variables considered clinically relevant were further assessed using a multivariable Cox proportional hazards regression model, and hazard ratios (HRs) with corresponding 95% CIs were reported. The proportional hazards assumption was evaluated by introducing time-dependent interaction terms between covariates and follow-up time. A statistically significant time-dependent interaction was considered evidence of a violation of the proportional hazards assumption. All statistical tests were two-sided, and a *P* value <.05 was considered statistically significant.

## 3. Results

A total of 307 patients with chronic hepatitis B were included in the study. The mean age was 50.2 ± 12.6 years, and the median follow-up duration was 77 months (range, 6–144 months). Histopathological evaluation revealed a median fibrosis stage of 2 (range, 1–5), a median HAI of 6 (range, 3–16), and a median histologic steatosis percentage of 0% (range, 0–60%).

The mean hematocrit level was 42.6 ± 5.1%, while the median hemoglobin concentration was 14.2 g/dL (range, 8.1–18.6). Median neutrophil, lymphocyte, and platelet counts were 3830/mm^3^, 1980/mm^3^, and 228,000/mm^3^, respectively. Median biochemical parameters included glucose 92 mg/dL, albumin 4.30 g/dL, total bilirubin 0.70 mg/dL, ALT 29 U/L, AST 28 U/L, ALP 72 U/L, GGT 19 U/L, AFP 2.9 ng/mL, urea 29 mg/dL, and creatinine 0.73 mg/dL.

Regarding lipid parameters, median total cholesterol, low-density lipoprotein cholesterol, high-density lipoprotein cholesterol, and triglyceride levels were 179 mg/dL, 109 mg/dL, 45.2 mg/dL, and 103 mg/dL, respectively. The overall median baseline log_10_ HBV DNA level was 4.09 (range, 0.42–9.17) IU/mL. Median HBV DNA levels decreased to 0 IU/mL at 6 months (range, 0–343,726,981 IU/mL), 12 months (range, 0–180,637 IU/mL), and 24 months (range, 0–13,189 IU/mL). The median time to HBV DNA suppression was 6 months (range, 6–60 months) (Table 1).

**Table 1:** Baseline demographic, histopathological, laboratory, and virological characteristics of patients with chronic hepatitis B (n = 307).

Variable	Value
Demographic characteristics	
Age (yr)	50.2 ± 12.6
Follow-up duration (mo)	77 (6–144)
Histopathological findings	
Histologic fibrosis stage	2 (1–5)
Histologic activity index (HAI)	6 (3–16)
Histologic steatosis (%)	0 (0–60)
Hematological parameters	
Hemoglobin (g/dL)	14.2 (8.1–18.6)
Hematocrit (%)	42.6 ± 5.1
Neutrophil count (/mm^3^)	3830 (390–12,470)
Lymphocyte count (/mm^3^)	1980 (170–4950)
Platelet count (/mm^3^)	228,000 (21,100–453,000)
Biochemical and liver function parameters	
Glucose (mg/dL)	92 (58–313)
Albumin (g/dL)	4.30 (0.10–5.30)
Total bilirubin (mg/dL)	0.70 (0.16–7.40)
ALT (U/L)	29 (3–2729)
AST (U/L)	28 (8–3155)
ALP (U/L)	72 (14–295)
GGT (U/L)	19 (2–707)
AFP (ng/mL)	2.9 (0–46)
Urea (mg/dL)	29 (0.8–267)
Creatinine (mg/dL)	0.73 (0.20–6.60)
Lipid profile	
Total cholesterol (mg/dL)	179 (36–367)
LDL cholesterol (mg/dL)	109 (0–270)
HDL cholesterol (mg/dL)	45.2 (10–114)
Triglycerides (mg/dL)	103 (30–7174)
Virological parameters	
Baseline HBV DNA (log_10_ IU/mL)	4.09 (0.42–9.17)
HBV DNA at 6 mo (IU/mL)	<10 (<10–343,726,981)
HBV DNA at 12 mo (IU/mL)	<10 (<10–180,637)
HBV DNA at 24 mo (IU/mL)	<10 (<10–13,18)
Outcome	
Time to HBV DNA suppression (mo)	6 (6–60)

Data are presented as mean ± standard deviation or median (minimum–maximum), as appropriate.

Continuous variables are expressed as mean ± standard deviation or median (range), according to distribution.

AFP = alpha-fetoprotein, ALP = alkaline phosphatase, ALT = alanine aminotransferase, AST = aspartate aminotransferase, GGT = gamma-glutamyl transferase, HAI = histologic activity index, HBV = hepatitis B virus, HBV DNA = hepatitis B virus deoxyribonucleic acid, HDL = high-density lipoprotein, IU = international units, LDL = low-density lipoprotein.

Among the 307 patients, 164 (53.4%) were male and 143 (46.6%) were female. Antiviral therapy consisted of tenofovir in 217 patients (70.7%) and entecavir in 90 patients (29.3%). All patients were HBsAg-positive, while 53 (17.3%) were HBeAg-positive and 245 of 305 patients (80.3%) were anti-HBe positive. Ultrasonographic hepatic steatosis was present in 102 patients (33.2%) and absent in 205 patients (66.8%). Assessment of liver echotexture demonstrated a normal pattern in 159 patients (51.8%), a fine granular pattern in 112 patients (36.5%), and a coarse granular pattern in 36 patients (11.7%). Liver contour was normal in 204 patients (66.4%), irregular in 89 patients (29.0%), and nodular in 14 patients (4.6%). No patient had hepatocellular carcinoma at baseline (Table 2).

**Table 2:** Baseline categorical characteristics of patients with chronic hepatitis B (n = 307).

Variable	n (%)
Sex	
Male	164 (53.4)
Female	143 (46.6)
Antiviral treatment	
Tenofovir	217 (70.7)
Entecavir	90 (29.3)
HBsAg positivity	307 (100.0)
HBeAg positivity	53 (17.3)
Anti-HBe positivity	245/305 (80.3)
Ultrasonographic steatosis	
Absent	205 (66.8)
Present	102 (33.2)
Liver echotexture	
Normal	159 (51.8)
Fine granular	112 (36.5)
Coarse granular	36 (11.7)
Liver contour	
Normal	204 (66.4)
Irregular	89 (29.0)
Nodular	14 (4.6)
HCC	0 (0.0)

Categorical variables are presented as number (percentage).

Anti-HBe = antibody to hepatitis B e antigen, HBeAg = hepatitis B e antigen, HBsAg = hepatitis B surface antigen, HCC = hepatocellular carcinoma.

Patients who achieved HBV DNA suppression at 12 months and those who did not were comparable with respect to age, follow-up duration, hematological parameters, glucose levels, liver function tests, renal function parameters, lipid profile, and AFP levels (all *P* > .05). Among laboratory variables, AST levels were significantly higher in patients who failed to achieve HBV DNA suppression at 12 months compared with those who achieved suppression (median 38 U/L vs 27 U/L, *P* = .024). Histopathological assessment demonstrated that patients without virological suppression had higher HAI scores (median 8 vs 6, *P* = .018) and greater histologic steatosis percentages (median 0.1% vs 0%, *P* = .006). Baseline log_10_ HBV DNA levels were significantly higher in patients who failed to achieve HBV DNA suppression at 12 months than in those who achieved virological suppression (4.62 [3.33–9.14] vs 4.07 [0.42–9.17], respectively; *P* = .022). Histologic fibrosis stage showed a trend toward significance but did not reach statistical significance (*P* = .076). No significant differences were observed in ALT, ALP, GGT, bilirubin, albumin, creatinine, cholesterol parameters, triglycerides, or time to HBV DNA suppression between the two groups (all *P* > .05) (Table 3) (Table S3, Supplemental Digital Content 1).

**Table 3:** Baseline characteristics according to 12-month event status.

Variable	HBV DNA not suppressed (n = 49)	HBV DNA suppressed (n = 258)	*P* value
Age (yr)	50.7 ± 14.4	49.9 ± 12.2	.719*
Follow-up duration (mo)	72 (36–144)	74 (6–144)	.904†
AFP (ng/mL)	2.9 (0.3–10.9)	2.9 (0–46)	.743†
Hemoglobin (g/dL)	14.2 ± 1.7	14.1 ± 1.8	.785†
Hematocrit (%)	42.3 ± 4.7	42.7 ± 5.1	.657*
Neutrophil count (/mm^3^)	4050 (1510–8190)	3800 (390–12,470)	.234†
Lymphocyte count (/mm^3^)	2020 (740–3640)	1980 (170–4950)	.793†
Platelet count (×10^3^/mm^3^)	222 (69–369)	229 (65–453)	.605†
Glucose (mg/dL)	88 (64–207)	93 (58–313)	.113†
Total bilirubin (mg/dL)	0.70 (0.20–7.00)	0.60 (0.16–7.40)	.243†
Albumin (g/dL)	4.30 (3.10–4.90)	4.30 (0.10–5.30)	.949†
ALT (U/L)	53 (13–343)	28 (3–2729)	.092†
AST (U/L)	38 (16–301)	27 (8–3155)	**.**024†
ALP (U/L)	71.5 (44–141)	72 (14–295)	.425†
GGT (U/L)	19.5 (8–337)	19 (2–707)	.483†
Urea (mg/dL)	31 (16–70)	28 (0.8–267)	.071†
Creatinine (mg/dL)	0.75 (0.36–1.29)	0.70 (0.20–6.60)	.399†
Total cholesterol (mg/dL)	181 (36–261)	179 (116–367)	.708†
LDL cholesterol (mg/dL)	107.1 ± 19.9	111.5 ± 36.6	.334*
HDL cholesterol (mg/dL)	48 (32–77)	45 (10–114)	.563†
Triglycerides (mg/dL)	97 (35–289)	104 (30–7174)	.809†
Baseline log_10_ HBV DNA (IU/mL)	4.62 (3.33–9.14)	4.07 (0.42–9.17)	.022†
Time to HBV DNA suppression (mo)	6 (6-36)	6 (6-60)	.399†
Histologic Activity Index (HAI)	8 (3–16)	6 (3–15)	.018†
Histologic steatosis (%)	0.1 (0–60)	0 (0–50)	.006†
Histologic fibrosis stage	2 (1–5)	2 (1–5)	.076†

AFP = alpha-fetoprotein, ALP = alkaline phosphatase, ALT = alanine aminotransferase, AST = aspartate aminotransferase, GGT = gamma-glutamyl transferase, HAI = histologic activity index, HDL = high-density lipoprotein, LDL = low-density lipoprotein.

Values are presented as mean ± standard deviation or median (range), as appropriate.

* Independent samples *t*-test.

† Mann–Whitney *U* test.

Analysis of categorical variables demonstrated that HBeAg status, liver parenchymal echotexture, liver contour, and histologic steatosis ≥5% were significantly associated with 12-month HBV DNA suppression. Patients who achieved virological suppression were more frequently HBeAg-negative than those who did not achieve suppression (84.9% vs 71.4%, *P* = .022). Although anti-HBe positivity was more common among patients with virological suppression (82.0% vs 71.4%), this difference did not reach statistical significance (*P* = .087). No significant associations were observed between 12-month HBV DNA suppression and sex (*P* = .956), antiviral treatment type (tenofovir versus entecavir; *P* = .640), or ultrasonographic steatosis (*P* = .917). Liver parenchymal echotexture differed significantly between groups (*P* < .001). Patients with virological suppression more frequently demonstrated a normal liver echotexture (57.4% vs 22.4%), whereas fine granular and coarse granular patterns were more common among patients without suppression. Similarly, liver contour was significantly associated with virological suppression (*P* < .001), with normal contour being more frequent in the suppression group (70.5% vs 44.9%), while irregular and nodular contours were more prevalent among patients who failed to achieve suppression. Histologic steatosis ≥5% was significantly more common in patients without virological suppression at 12 months than in those who achieved suppression (42.9% vs 23.6%, *P* = .005) (Table 4).

**Table 4:** Association between categorical variables and 12-month event status.

Variable	HBV DNA not suppressed (n = 49)	HBV DNA suppressed (n = 258)	*P* value
Sex, n (%)			.956
Male	26 (53.1)	138 (53.5)	
Female	23 (46.9)	120 (46.5)	
Antiviral treatment, n (%)			.640
Tenofovir	36 (73.5)	181 (70.2)	
Entecavir	13 (26.5)	77 (29.8)	
HBeAg status, n (%)			.022
Negative	35 (71.4)	219 (84.9)	
Positive	14 (28.6)	39 (15.1)	
Anti-HBe status, n (%)			.087
Negative	14 (28.6)	46 (18.0)	
Positive	35 (71.4)	210 (82.0)	
Ultrasonographic steatosis, n (%)			.917
Absent	34 (69.4)	171 (66.3)	
Present	15 (30.6)	87 (33.7)	
Liver parenchymal echotexture, n (%)			<.001
Normal	11 (22.4)	148 (57.4)	
Fine granular	29 (59.2)	83 (32.2)	
Coarse granular	9 (18.4)	27 (10.5)	
Liver contour, n (%)			<.001
Normal	22 (44.9)	182 (70.5)	
Irregular	21 (42.9)	68 (26.4)	
Nodular	6 (12.2)	8 (3.1)	
Histologic steatosis ≥ 5%, n (%)			.005
No	28 (57.1)	197 (76.4)	
Yes	21 (42.9)	61 (23.6)	

Data are presented as n (%). Comparisons between groups were performed using Pearson’s chi-square test; Fisher’s exact test was used when expected cell counts were < 5.

Anti-HBe = antibody to hepatitis B e antigen, HBeAg = hepatitis B e antigen.

In the primary multivariable logistic regression analysis, histologic steatosis ≥5% and higher baseline log_10_ HBV DNA were associated with a lower likelihood of achieving HBV DNA suppression at 12 months. Patients with histologic steatosis ≥5% had lower odds of achieving virological suppression than those with steatosis <5% (OR 0.365, 95% CI 0.179–0.746; *P* = .006). Similarly, each 1-log_10_ increase in baseline HBV DNA was associated with lower odds of 12-month virological suppression (OR 0.842, 95% CI 0.751–0.944; *P* = .003). Age, sex, antiviral regimen, HBeAg status, AST level, HAI, and fibrosis stage were not significantly associated with 12-month virological suppression (all *P* > .05).

To assess the robustness of these findings while reducing the potential for overfitting, a parsimonious sensitivity model including histologic steatosis, baseline log_10_ HBV DNA, HBeAg status, and antiviral treatment was additionally evaluated. Histologic steatosis ≥5% remained significantly associated with a lower likelihood of 12-month HBV DNA suppression (OR 0.398, 95% CI 0.206–0.767; *P* = .006), as did higher baseline log_10_ HBV DNA (OR 0.870, 95% CI 0.787–0.962; *P* = .007). The similarity of the effect estimates between the primary and parsimonious models supported the robustness of these associations (Table 5) (Table S1–S2, Supplemental Digital Content 2).

**Table 5:** Primary and parsimonious multivariable logistic regression models for HBV DNA suppression at 12 months.

Variable	Primary model OR (95% CI)	*P* value	Parsimonious model OR (95% CI)	*P* value
Age (yr)	0.983 (0.955–1.012)	.254	–	–
Male sex	1.537 (0.765–3.086)	.227	–	–
Tenofovir treatment	1.181 (0.561–2.488)	.661	1.241 (0.596–2.587)	.564
HBeAg positivity	1.116 (0.402–3.095)	.833	1.216 (0.449–3.291)	.700
AST (U/L)	1.005 (0.997–1.012)	.213	–	–
Histologic activity index (HAI)	1.036 (0.827–1.298)	.761	–	–
Histologic steatosis ≥ 5%	0.365 (0.179–0.746)	.006	0.398 (0.206–0.767)	.006
Fibrosis stage	0.831 (0.507–1.361)	.462	–	–
Baseline log_10_ HBV DNA (IU/mL)	0.842 (0.751–0.944)	.003	0.870 (0.787–0.962)	.007

ORs and corresponding 95% CIs are reported for the primary and parsimonious multivariable logistic regression models evaluating HBV DNA suppression at 12 months.

AST = aspartate aminotransferas, CI = confidence interval, HAI = histologic activity index, OR = odds ratio.

Kaplan–Meier analysis demonstrated a significant difference in time to HBV DNA suppression according to histologic steatosis status. Patients with histologic steatosis ≥5% experienced delayed virological suppression compared with those with steatosis < 5%. The median time to HBV DNA suppression was 12 months in patients with steatosis ≥5% and 6 months in those with steatosis <5%. Log-rank analysis confirmed a significant difference between the two groups (χ^2^ = 5.842, *P* = .016) (Fig. 1).

**Figure 1.**
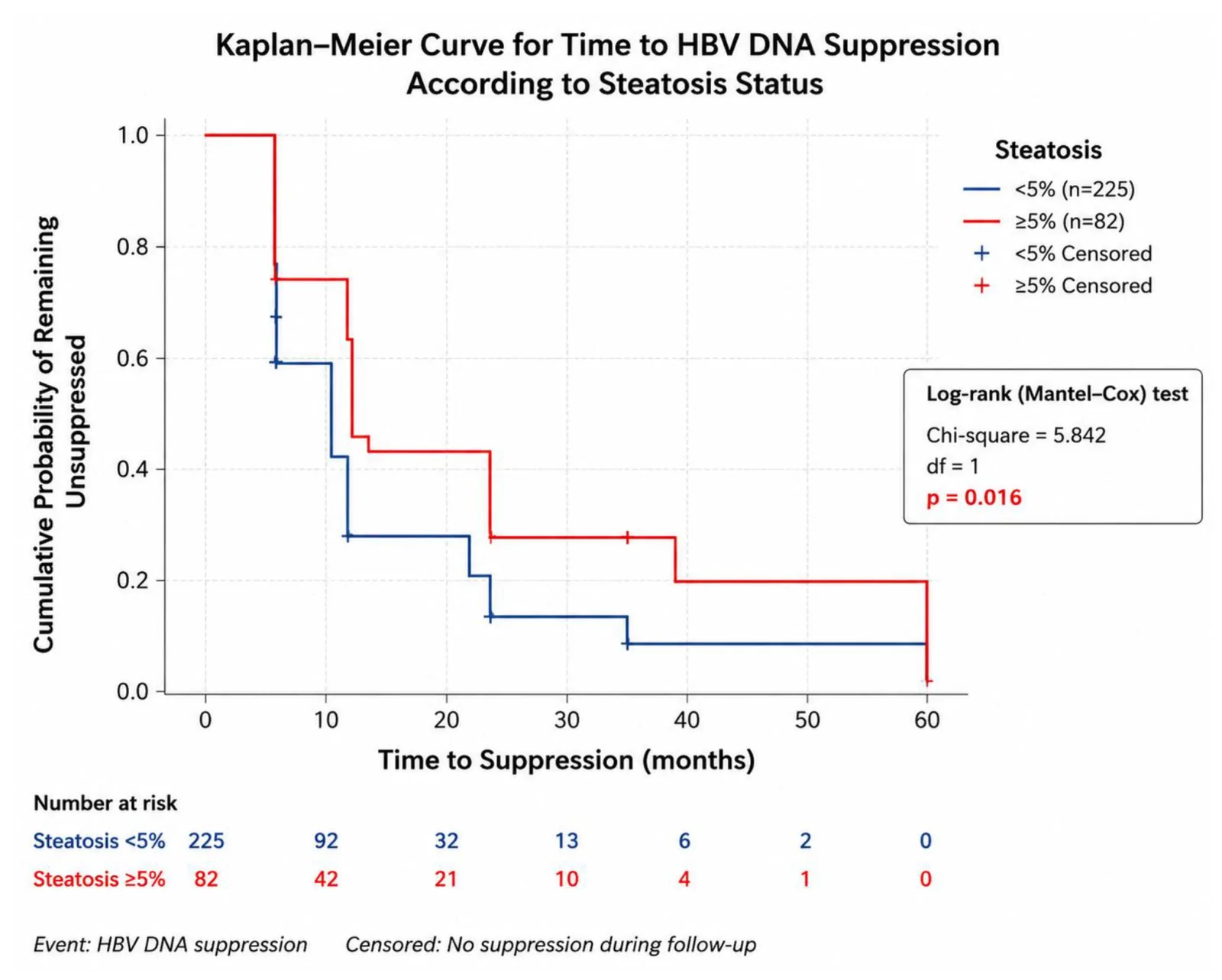
Kaplan–Meier analysis of time to HBV DNA suppression according to hepatic steatosis status. Kaplan–Meier curves show the cumulative probability of remaining without HBV DNA suppression during follow-up in patients with hepatic steatosis < 5% and ≥ 5%. Patients with hepatic steatosis ≥ 5% demonstrated a significantly longer time to virological suppression compared with those with steatosis < 5% (log-rank [Mantel–Cox] test: *χ*^*2*^ = 5.842, df = 1, *P* = .016). Censoring marks indicate patients who did not achieve HBV DNA suppression during the available follow-up period. Numbers at risk are shown below the graph. HBV = hepatitis B virus. HBeAg = hepatitis B e antigen, HBV = hepatitis B virus,

Kaplan–Meier analysis demonstrated a significant association between HBeAg status and time to HBV DNA suppression. Patients who were HBeAg-positive experienced delayed virological suppression compared with HBeAg-negative patients. The median time to HBV DNA suppression was 12 months in HBeAg-positive patients and 6 months in HBeAg-negative patients. Log-rank analysis confirmed a significant difference between the two groups (*χ*^*2*^ = 7.940, *P* = .005) (Fig. 2).

**Figure 2.**
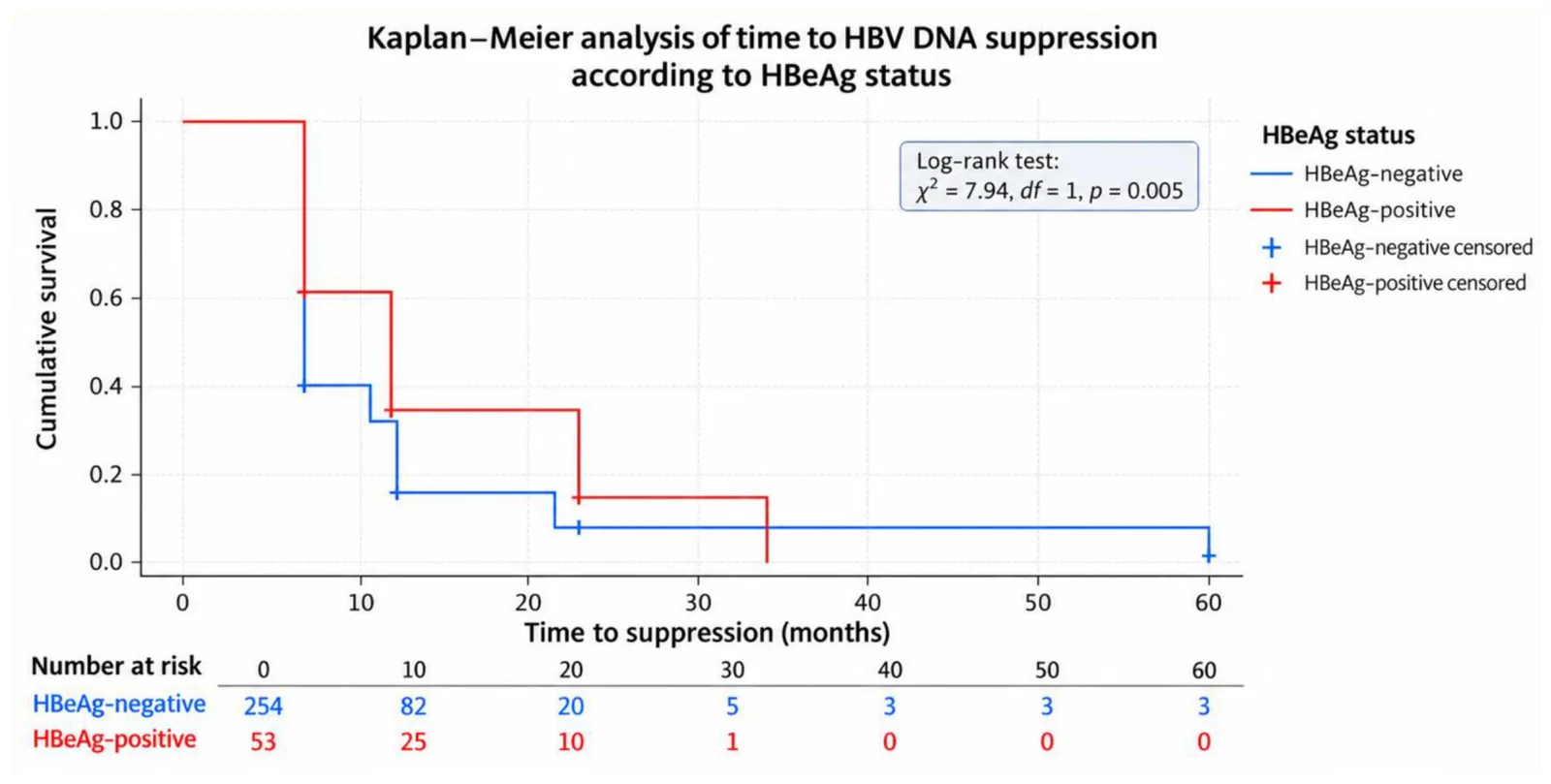
Kaplan–Meier analysis of time to HBV DNA suppression according to HBeAg status. Kaplan–Meier curves demonstrate the cumulative probability of remaining without HBV DNA suppression during follow-up in HBeAg-negative and HBeAg-positive patients. HBeAg-positive patients had a significantly longer time to virological suppression compared with HBeAg-negative patients (log-rank test: *χ*^*2*^ = 7.94, df = 1, *P* = .005). Censoring marks indicate patients who did not achieve HBV DNA suppression during the available follow-up period. Numbers at risk are presented below the graph. HBeAg = hepatitis B e antigen, HBV = hepatitis B virus.

Kaplan–Meier analysis demonstrated that HBV DNA suppression occurred rapidly during follow-up. The median time to virological suppression was 6 months, indicating that more than half of the patients achieved HBV DNA suppression within the first 6 months of treatment. The estimated mean time to suppression was 13.2 months (95% CI: 11.4–15.0 months). The cumulative probability of remaining HBV DNA positive decreased markedly during the first year of follow-up, with a smaller proportion of patients achieving suppression thereafter. All patients achieved HBV DNA suppression by 60 months of follow-up (Fig. 3).

**Figure 3.**
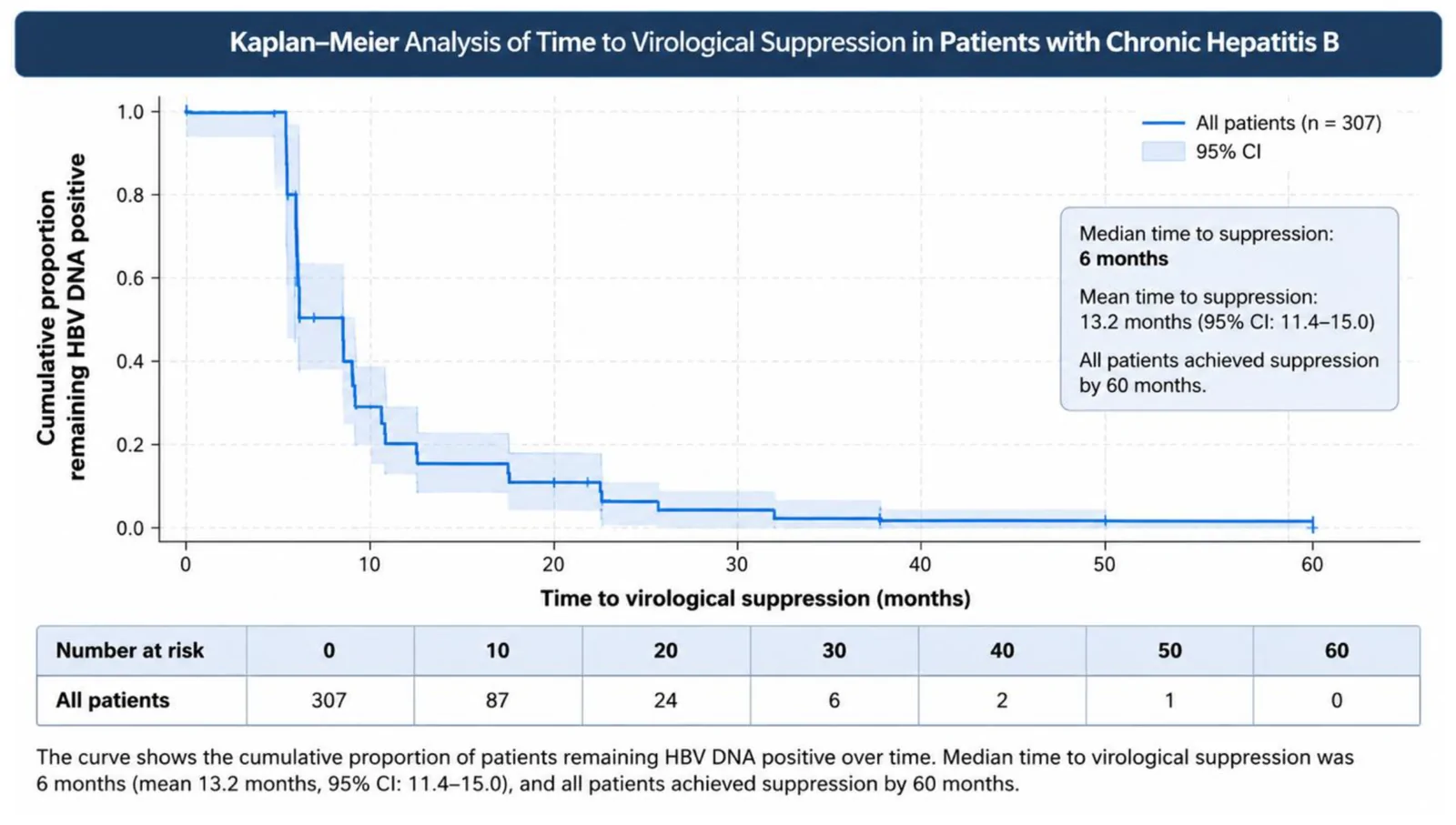
Kaplan–Meier analysis of time to virological suppression in patients with chronic hepatitis B. The Kaplan–Meier curve shows the cumulative proportion of patients remaining HBV DNA positive during follow-up. The median time to virological suppression was 6 months, while the mean time to suppression was 13.2 months (95% CI, 11.4–15.0 months). All patients achieved virological suppression by 60 months. Shaded areas represent the 95% confidence interval, censoring marks indicate censored observations, and numbers at risk are shown below the graph. HBV = hepatitis B virus.

Multivariable Cox proportional hazards regression analysis was performed to identify independent predictors of time to virological suppression. After adjustment for age, sex, antiviral treatment, HBeAg status, histologic steatosis, fibrosis stage, HAI, and baseline log_10_ HBV DNA level, none of the evaluated variables remained independently associated with the time to virological suppression. Specifically, age (HR, 1.005; 95% CI, 0.995–1.015; *P* = .352), male sex (HR, 1.055; 95% CI, 0.830–1.341; *P* = .662), treatment with tenofovir versus entecavir (HR, 1.085; 95% CI, 0.844–1.394; *P* = .524), HBeAg positivity (HR, 1.020; 95% CI, 0.699–1.490; *P* = .917), histologic steatosis ≥5% (HR, 0.900; 95% CI, 0.689–1.175; *P* = .438), fibrosis stage (HR, 1.010; 95% CI, 0.849–1.200; *P* = .914), HAI (HR, 0.992; 95% CI, 0.921–1.068; *P* = .824), and baseline log_10_ HBV DNA level (HR, 0.945; 95% CI, 0.865–1.033; *P* = .214) were not significant independent predictors (Table 6). The proportional hazards assumption was additionally examined using time-dependent covariate analysis. A significant interaction between histologic steatosis and follow-up time was observed (*P* < .001), indicating that the association between histologic steatosis and the rate of virological suppression was not constant over time. Therefore, the overall hazard ratio for histologic steatosis derived from the conventional Cox model should be interpreted as an average effect across follow-up rather than as a constant effect over time.

**Table 6:** Cox proportional hazards analysis for predictors of HBV DNA suppression.

Variable	*β*	SE	HR (95% CI)	*P* value
Age	0.005	0.005	1.005 (0.995–1.015)	.352
Male sex	0.054	0.122	1.055 (0.830–1.341)	.662
Tenofovir vs Entecavir	0.082	0.128	1.085 (0.844–1.394)	.524
HBeAg positive	0.020	0.193	1.020 (0.699–1.490)	.917
Steatosis ≥ 5%	−0.106	0.136	0.900 (0.689–1.175)	.438
Fibrosis stage	0.009	0.088	1.010 (0.849–1.200)	.914
Histologic Activity Index (HAI)	−0.008	0.038	0.992 (0.921–1.068)	.824
Baseline log_10_ HBV DNA (IU/mL)	−0.056	0.045	0.945 (0.865–1.033)	.214

HR >1 indicates a higher rate of HBV DNA suppression, whereas HR <1 indicates a lowe rate of suppression. Cox proportional hazards regression analysis for predictors of HBV DNA suppression.

CI = confidence interval, HAI = histologic activity index, HBeAg = hepatitis B e antigen, HR = hazard ratio, SE = standard error.

## 4. Discussion

In the present study, we evaluated the impact of histologic hepatic steatosis on virological response in patients with chronic hepatitis B receiving tenofovir or entecavir therapy. Histologic steatosis ≥5% was independently associated with a lower likelihood of achieving HBV DNA suppression at 12 months and with a longer time to virological suppression in Kaplan–Meier analysis. In addition, higher baseline log_10_ HBV DNA levels independently predicted reduced 12-month virological suppression. However, neither histologic steatosis nor baseline viral load remained an independent predictor of time to HBV DNA suppression in the multivariable Cox regression model. These findings suggest that hepatic steatosis may primarily influence the timing of early virological response rather than the ultimate long-term effectiveness of antiviral therapy.

The relationship between chronic hepatitis B and hepatic steatosis has been extensively investigated; however, the available evidence remains inconclusive. While some studies have suggested that hepatic steatosis may suppress HBV replication and facilitate HBsAg loss, others have reported that steatosis is associated with accelerated liver disease progression and a less favorable response to antiviral therapy.^[10–12]^ In the present study, the observed association between histologic steatosis and a reduced early virological response is consistent with previous reports indicating that hepatic steatosis may adversely affect the efficacy of antiviral treatment.^[11,12]^

The mechanisms underlying this association have not yet been fully elucidated. Hepatic steatosis is frequently accompanied by obesity, insulin resistance, metabolic syndrome, and type 2 diabetes mellitus, which may influence HBV-related outcomes through chronic inflammation, oxidative stress, mitochondrial dysfunction, and altered intrahepatic immune responses.^[13–15]^ Lipid accumulation may also affect intracellular signaling and antiviral efficacy.^[13,14]^ Beyond direct hepatic fat accumulation, systemic and metabolic factors may contribute to heterogeneity in HBV-related outcomes. Sex-related disparities in viral hepatitis burden have been reported, potentially reflecting differences in hormonal, immune, metabolic, and behavioral factors.^[16]^ Systemic inflammatory activity may also influence host–virus interactions, and complete blood count-derived indices have emerged as accessible markers of inflammatory and immune status.^[17]^ Oxidative imbalance may further contribute through increased cellular oxidative stress and impaired antioxidant defenses.^[18]^ In addition, interactions between lipid metabolism and immune regulation may influence inflammatory signaling and host immune responses, providing another potential link between metabolic alterations and disease outcomes.^[19]^ Although these findings do not establish these mechanisms specifically in chronic hepatitis B, they provide a broader biological context for the present findings. Given that complete blood count and lipid parameters were available in our cohort, future studies could investigate whether readily available inflammatory and lipid-related markers improve risk stratification for virological response. Comprehensive metabolic data were not available in the present study; therefore, the potential contribution of underlying metabolic dysfunction requires further investigation.

One of the principal findings of the present study was the differential association of histologic steatosis with early and longer-term virological outcomes. Histologic steatosis was associated with a lower likelihood of HBV DNA suppression at 12 months in the multivariable logistic regression model, whereas Kaplan–Meier analysis demonstrated a significantly longer time to virological suppression among patients with histologic steatosis. In the conventional multivariable Cox model, however, histologic steatosis was not significantly associated with time to suppression. Further assessment demonstrated a significant interaction between histologic steatosis and follow-up time (*P* < .001), indicating a violation of the proportional hazards assumption for steatosis and suggesting that its association with virological suppression varied during follow-up. This time-dependent pattern may help explain the apparent discrepancy between the significant 12-month logistic regression findings and the nonsignificant overall hazard ratio from the conventional Cox model. Previous systematic reviews and meta-analyses have similarly suggested that the association between hepatic steatosis and antiviral treatment response in chronic hepatitis B is heterogeneous and may vary according to patient characteristics, methods used to assess steatosis, and accompanying metabolic risk factors.^[11,12]^ Together, these findings suggest that the association between hepatic steatosis and virological response may be more apparent during the earlier treatment period; however, the present analysis does not establish a specific time point at which this association attenuates or disappears.

Higher baseline HBV DNA has consistently been recognized as one of the strongest predictors of delayed virological response during nucleos(t)ide analogue therapy. Patients with higher pretreatment viral loads generally require a longer duration of antiviral treatment to achieve complete viral suppression because of a larger intrahepatic viral reservoir and sustained viral replication.^[20,21]^ Consistent with previous studies, higher baseline log_10_ HBV DNA levels independently predicted reduced 12-month virological suppression in the present study. However, this association was not maintained in the multivariable Cox regression analysis, suggesting that baseline viral load primarily influences the timing of early virological response rather than the likelihood of achieving long-term viral suppression.

In the present study, ultrasonographically detected steatosis was not significantly associated with virological response, whereas histologic steatosis demonstrated a significant association with treatment outcomes. Given the limited sensitivity of ultrasonography for detecting mild hepatic steatosis, particularly when the extent of fat accumulation is low, these findings suggest that histologic assessment may provide greater prognostic value in predicting early virological response. This observation is consistent with the established role of liver biopsy as the reference standard for the assessment and grading of hepatic steatosis.^[9]^

In the univariable analysis, patients who failed to achieve HBV DNA suppression at 12 months had higher AST levels and HAI scores than those who achieved virological suppression. However, neither AST nor HAI remained significantly associated with virological response after adjustment for baseline log_10_ HBV DNA or in multivariable analyses. In contrast, the association between histologic steatosis and reduced 12-month virological suppression remained significant after adjustment for baseline viral load, suggesting that histologic steatosis may represent a more robust predictor of early treatment response than histologic inflammatory activity.^[22]^

This study has several important strengths. First, it included a relatively large cohort of patients, all of whom underwent pretreatment liver biopsy, allowing the use of histologically confirmed hepatic steatosis rather than imaging-based assessment. Second, both short-term (12-month HBV DNA suppression) and long-term (time to virological suppression) virological outcomes were evaluated, providing a comprehensive assessment of antiviral treatment response. Finally, the relatively long follow-up period enabled a detailed evaluation of the temporal characteristics of virological response during nucleos(t)ide analogue therapy.

This study also has several limitations. First, its retrospective, single-center design introduces the potential for selection bias and may limit the generalizability of the findings. Second, important metabolic variables associated with hepatic steatosis, including body mass index, insulin resistance, diabetes mellitus, and other metabolic risk factors, were not available for all patients and therefore could not be incorporated into the multivariable analyses. Consequently, residual confounding cannot be completely excluded, and the observed association between histologic steatosis and virological response cannot be fully separated from the potential influence of underlying metabolic dysfunction. Third, liver biopsy was performed only at baseline; therefore, dynamic changes in hepatic steatosis during antiviral therapy could not be evaluated. In addition, liver biopsy is subject to sampling variability and may not fully represent the overall hepatic fat content or inflammatory activity. Finally, although antiviral treatment type was adjusted for in the multivariable analyses, treatment allocation was not randomized. Because of the observational nature of the study, causal relationships between hepatic steatosis and virological response cannot be established.

## 5. Conclusion

In conclusion, histologic hepatic steatosis ≥5% and higher baseline log_10_ HBV DNA levels were associated with a lower likelihood of achieving HBV DNA suppression at 12 months after adjustment for available demographic, virological, and histopathological factors. Histologic steatosis was also associated with delayed time to virological suppression in unadjusted Kaplan–Meier analysis. However, neither histologic steatosis nor baseline HBV DNA remained independently associated with time to HBV DNA suppression in the multivariable Cox regression model. These findings suggest that hepatic steatosis may be associated with the early virological response, whereas an independent association with longitudinal time to suppression was not demonstrated. Nevertheless, because comprehensive metabolic data, including BMI, diabetes status, and insulin resistance, were unavailable, residual confounding by underlying metabolic dysfunction cannot be excluded. Therefore, the observed association should not be interpreted as demonstrating a causal effect of hepatic steatosis per se.

## Author contributions

**Conceptualization:** Sedat Çiçek, Jehat Kiliç, Mehmet Yalniz.

**Investigation:** Sedat Çiçek, Jehat Kiliç, Abdullah Mübin Özercan, Abdulvahap Hohluoğlu, Furkan Kirsoy, Selman Çetin, Ali Çağri Oral.

**Methodology:** Sedat Çiçek, Jehat Kiliç, İbrahim Halil Bahçecioğlu.

**Project administration:** Sedat Çiçek.

**Resources:** Sedat Çiçek, Mehmet Yalniz, İbrahim Halil Bahçecioğlu.

**Writing – review & editing:** Sedat Çiçek, Abdullah Mübin Özercan, Abdulvahap Hohluoğlu, İbrahim Hanifi Özercan, Mehmet Yalniz, İbrahim Halil Bahçecioğlu.

**Data curation:** Jehat Kiliç, Furkan Kirsoy, Selman Çetin, Ali Çağri Oral.

**Formal analysis:** Jehat Kiliç.

**Visualization:** Jehat Kiliç.

**Writing – original draft:** Jehat Kiliç.

**Supervision:** Mehmet Yalniz, İbrahim Halil Bahçecioğlu.






