# Supplementary material for: Impact of histologic hepatic steatosis on virological response to nucleos(t)ide analogue therapy in patients with chronic hepatitis B
Source: Medicine (Baltimore). 2026 Oct 9;105(41):e51074. doi: 10.1097/MD.0000000000051074 (PMC13657455; doi:10.1097/MD.0000000000051074)
Supplement: Supplementary file 1 [file medi-105-e51074-s001.docx]

**Supplementary Table S3.** Sensitivity analysis using analysis of covariance (ANCOVA) adjusting for baseline log₁₀ HBV DNA

| Dependent variable | Predictor | F | p value |
| --- | --- | --- | --- |
| ****AST (U/L)**** | Baseline log₁₀ HBV DNA | 0.070 | 0.791 |
|  | 12-month HBV DNA suppression | 0.236 | 0.627 |
| ****Histologic steatosis (%)**** | Baseline log₁₀ HBV DNA | 1.568 | 0.212 |
|  | 12-month HBV DNA suppression | **9.436** | **0.002** |
| ****Histologic Activity Index (HAI)**** | Baseline log₁₀ HBV DNA | 0.870 | 0.352 |
|  | 12-month HBV DNA suppression | 2.545 | 0.112 |
| ****HBeAg positivity**** | Baseline log₁₀ HBV DNA | **51.531** | **<0.001** |
|  | 12-month HBV DNA suppression | 0.719 | 0.397 |

**Abbreviations:** AST, aspartate aminotransferase; HAI, Histologic Activity Index; HBeAg, hepatitis B e antigen; HBV, hepatitis B virus.
