# Supplementary material for: Impact of histologic hepatic steatosis on virological response to nucleos(t)ide analogue therapy in patients with chronic hepatitis B
Source: Medicine (Baltimore). 2026 Oct 9;105(41):e51074. doi: 10.1097/MD.0000000000051074 (PMC13657455; doi:10.1097/MD.0000000000051074)
Supplement: Supplementary file 2 [file medi-105-e51074-s002.docx]

**Supplementary Table S1:** Performance characteristics of the multivariable logistic regression model predicting HBV DNA suppression at 12 months

| Parameter | Value |
| --- | --- |
| Hosmer–Lemeshow χ² | 11.826 |
| Degrees of freedom | 8 |
| Hosmer–Lemeshow p-value | 0.159 |
| Area under ROC curve (AUC) | 0.688 |
| Model calibration | Acceptable |
| Model discrimination | Modest |

**Abbreviations:** AUC, area under the receiver operating characteristic curve; HBV, hepatitis B virus; ROC, receiver operating characteristic.
