# Supplementary material for: Impact of histologic hepatic steatosis on virological response to nucleos(t)ide analogue therapy in patients with chronic hepatitis B
Source: Medicine (Baltimore). 2026 Oct 9;105(41):e51074. doi: 10.1097/MD.0000000000051074 (PMC13657455; doi:10.1097/MD.0000000000051074)
Supplement: Supplementary file 3 [file medi-105-e51074-s003.docx]

**Supplementary Table S2.** Median time to HBV DNA suppression according to HBeAg and histologic steatosis status

| Variable | Group | Median time to HBV DNA suppression |
| --- | --- | --- |
| HBeAg status | HBeAg negative | **6 months** |
|  | HBeAg positive | **12 months** |
| Histologic steatosis | <5% | **6 months** |
|  | ≥5% | **12 months** |

**Abbreviations:** HBeAg, hepatitis B e antigen; HBV, hepatitis B virus.
